# *PATZ1* fusions define a novel molecularly distinct neuroepithelial tumor entity with a broad histological spectrum

**DOI:** 10.1007/s00401-021-02354-8

**Published:** 2021-08-21

**Authors:** Karam T. Alhalabi, Damian Stichel, Philipp Sievers, Heike Peterziel, Alexander C. Sommerkamp, Dominik Sturm, Andrea Wittmann, Martin Sill, Natalie Jäger, Pengbo Beck, Kristian W. Pajtler, Matija Snuderl, George Jour, Michael Delorenzo, Allison M. Martin, Adam Levy, Nagma Dalvi, Jordan R. Hansford, Nicholas G. Gottardo, Emmanuelle Uro-Coste, Claude-Alain Maurage, Catherine Godfraind, Fanny Vandenbos, Torsten Pietsch, Christof Kramm, Maria Filippidou, Antonis Kattamis, Chris Jones, Ingrid Øra, Torben Stamm Mikkelsen, Michal Zapotocky, David Sumerauer, David Scheie, Martin McCabe, Pieter Wesseling, Bastiaan B. J. Tops, Mariëtte E. G. Kranendonk, Matthias A. Karajannis, Nancy Bouvier, Elli Papaemmanuil, Hildegard Dohmen, Till Acker, Katja von Hoff, Simone Schmid, Evelina Miele, Katharina Filipski, Lidija Kitanovski, Lenka Krskova, Johannes Gojo, Christine Haberler, Frank Alvaro, Jonas Ecker, Florian Selt, Till Milde, Olaf Witt, Ina Oehme, Marcel Kool, Andreas von Deimling, Andrey Korshunov, Stefan M. Pfister, Felix Sahm, David T. W. Jones

**Affiliations:** 1 Hopp Children’s Cancer Center Heidelberg (KiTZ), Heidelberg, Germany; 2 Division of Pediatric Glioma Research (B360), German Cancer Research Center (DKFZ), Im Neuenheimer Feld 280, 69120 Heidelberg, Germany; 3 Faculty of Medicine, Heidelberg University, Heidelberg, Germany; 4 Department of Neuropathology, Heidelberg University Hospital, Heidelberg, Germany; 5 Clinical Cooperation Unit Neuropathology, German Consortium for Translational Cancer Research (DKTK), German Cancer Research Center (DKFZ), Heidelberg, Germany; 6 Division of Pediatric Neurooncology, German Cancer Research Center (DKFZ), Heidelberg, Germany; 7 Department of Pediatric Oncology, Hematology and Immunology, Heidelberg University Hospital, Heidelberg, Germany; 8 Clinical Cooperation Unit Pediatric Oncology, and German Consortium for Translational Cancer Research (DKTK), German Cancer Research Center (DKFZ), Heidelberg, Germany; 9 Division of Neuropathology, NYU Langone Health, New York, NY USA; 10 Department of Pathology, NYU Langone Health, New York, NY USA; 11 Department of Pediatrics, Albert Einstein College of Medicine, Bronx, New York, USA; 12 Isabel Rapin Division of Child Neurology, Albert Einstein College of Medicine, Bronx, New York, USA; 13 Department of Pediatrics, Children’s Cancer Centre, Royal Children’s Hospital, Murdoch Children’s Research Institute, University of Melbourne, Melbourne, VIC Australia; 14 Department of Oncology and Haematology, Perth Children’s Hospital, Perth, WA Australia; 15 Department of Pathology, IUCT-Oncopole, Toulouse University Hospital, Toulouse, France; 16 INSERM U1037, Team 11, Cancer Research Center of Toulouse (CRCT), Toulouse, France; 17 Department of Pathology, Lille University Hospital, Lille, France; 18 INSERM U837 UMR-S1172, Centre de Recherche Jean Pierre Aubert, Team 1, Lille, France; 19 Laboratory of Pathology, University Hospital of Clermont-Ferrand, Clermont-Ferrand, France; 20 University Clermont-Auvergne, M2iSH UMR1071, Clermont-Ferrand, France; 21 Human Molecular Genetics, de Duve Institute, Université Catholique de Louvain, Brussels, Belgium; 22 Department of Pathology, CHU Gabriel Montpied, Clermont‐Ferrand, France; 23 Institute of Neuropathology, Brain Tumor Reference Center of the Society for Neuropathology and Neuroanatomy, University of Bonn Medical Center, Bonn, Germany; 24 Division of Pediatric Hematology and Oncology, University Medical Center Göttingen, Gottingen, Germany; 25 Division of Pediatric Hematology-Oncology, First Department of Pediatrics, National and Kapodistrian University of Athens, Athens, Greece; 26 Division of Molecular Pathology, Institute of Cancer Research, London, UK; 27 Children’s Hospital, Paediatric Oncology, Skåne University Hospital, Lund, Sweden; 28 Paediatric and Adolescent Medicine, Department of Clinical Medicine, Aarhus University Hospital, Aarhus, Denmark; 29 Department of Pediatric Hematology and Oncology, Second Faculty of Medicine, University Hospital Motol, Charles University, Prague, Czech Republic; 30 Department of Clinical Medicine, Rigshospitalet, University of Copenhagen, Copenhagen, Denmark; 31 Division of Cancer Sciences, University of Manchester, Manchester, UK; 32 Department of Pathology, Brain Tumor Center Amsterdam University Medical Center, Amsterdam Universities Medical Centers/VUmc, Amsterdam, The Netherlands; 33 Princess Maxima Center for Pediatric Oncology, Utrecht, The Netherlands; 34 University Medical Center Utrecht, Utrecht, The Netherlands; 35 Department of Pediatrics, Memorial Sloan Kettering Cancer Center, New York, USA; 36 Marie-Josée and Henry R. Kravis Center for Molecular Oncology, Memorial Sloan Kettering Cancer Center, New York, NY USA; 37 Computational Oncology Service, Department of Epidemiology and Biostatistics, Memorial Sloan Kettering Cancer Center, New York, NY USA; 38 Center for Hematologic Malignancies, Memorial Sloan Kettering Cancer Center, New York, NY USA; 39 Department of Neuropathology, University Giessen, Giessen, Germany; 40 Department of Pediatric Oncology and Hematology, Freie Universität Berlin, Humboldt-Universität Zu Berlin, and Berlin Institute of Health, Charité – Universitätsmedizin Berlin, Berlin, Germany; 41 Department of Oncology, Hematology, Cell Therapy, Gene Therapy and Haemopoietic Transplant, Bambino Gesù Children’s Hospital, IRCCS, Rome, Italy; 42 Neurological Institute (Edinger Institute), University Hospital, Frankfurt, Germany; 43 German Cancer Consortium (DKTK), German Cancer Research Center (DKFZ), Heidelberg, Germany; 44 Frankfurt Cancer Institute (FCI), Frankfurt, Germany; 45 Division of Paediatrics, Department of Haematooncology, University Medical Center Ljubljana, Ljubljana, Slovenia; 46 Department of Pathology and Molecular Medicine, Second Faculty of Medicine, Charles University, Prague, Czech Republic; 47 Department of Pediatrics and Adolescent Medicine and Comprehensive Center for Pediatrics, Medical University of Vienna, Vienna, Austria; 48 Division of Neuropathology and Neurochemistry, Department of Neurology, Medical University of Vienna, Vienna, Austria; 49 University of Newcastle, Newcastle, NSW Australia; 50 John Hunter Children’s Hospital Newcastle, Newcastle, NSW Australia

**Keywords:** Brain tumor, Pediatric, Neurooncology, Neuroepithelial, PATZ1, EWSR1, MN1, Gene fusion

## Abstract

Large-scale molecular profiling studies in recent years have shown that central nervous system (CNS) tumors display a much greater heterogeneity in terms of molecularly distinct entities, cellular origins and genetic drivers than anticipated from histological assessment. DNA methylation profiling has emerged as a useful tool for robust tumor classification, providing new insights into these heterogeneous molecular classes. This is particularly true for rare CNS tumors with a broad morphological spectrum, which are not possible to assign as separate entities based on histological similarity alone. Here, we describe a molecularly distinct subset of predominantly pediatric CNS neoplasms (*n* = 60) that harbor *PATZ1* fusions. The original histological diagnoses of these tumors covered a wide spectrum of tumor types and malignancy grades. While the single most common diagnosis was glioblastoma (GBM), clinical data of the *PATZ1*-fused tumors showed a better prognosis than typical GBM, despite frequent relapses. RNA sequencing revealed recurrent *MN1*:*PATZ1* or *EWSR1*:*PATZ1* fusions related to (often extensive) copy number variations on chromosome 22, where *PATZ1* and the two fusion partners are located. These fusions have individually been reported in a number of glial/glioneuronal tumors, as well as extracranial sarcomas. We show here that they are more common than previously acknowledged, and together define a biologically distinct CNS tumor type with high expression of neural development markers such as *PAX2*, *GATA2* and *IGF2*. Drug screening performed on the *MN1*:*PATZ1* fusion-bearing KS-1 brain tumor cell line revealed preliminary candidates for further study. In summary, *PATZ1* fusions define a molecular class of histologically polyphenotypic neuroepithelial tumors, which show an intermediate prognosis under current treatment regimens.

**Supplementary Information:**

The online version contains supplementary material available at 10.1007/s00401-021-02354-8.

## Introduction

Human central nervous system (CNS) tumors are an incredibly diverse set of neoplasms, reflecting the vast array of different temporo-spatially distinct stem/progenitor cells that are present (and which may undergo oncogenic transformation) at different stages of development. Recently, DNA methylation-based classification of CNS tumors has been shown to be a robust tool for molecular tumor classification, likely on the basis that individual tumor types maintain an epigenetic ‘memory’ of their distinct cell of origin [5]. Over the past years, many studies have confirmed the prognostic relevance of such profiling, and its ability to provide a molecular stratification with notable clinical and biological correlates across diverse CNS tumor entities (e.g. [5, 19, 31, 40]). Such an approach is particularly valuable for the identification of molecular subgroups displaying a broad morphological spectrum, which may not have previously been identified as a distinct entity due to their rarity or lack of obvious unifying features. This principle was recently demonstrated with the identification of four new molecular tumor types within the morphologically heterogeneous group previously referred to as CNS primitive neuroectodermal tumor (PNET) [46].

This substantial heterogeneity also extends to the glioma family. Until recently, malignant gliomas in children (pediatric high-grade glioma, pedHGG) were considered similar to their adult glioblastoma counterparts. The discovery of a variety of pediatric-enriched alterations, such as histone 3 mutations (K27M and G34R/V), *PDGFRA* alterations and others, has substantially increased our understanding of the molecular background of pedHGG [15, 32, 41, 55]. In parallel, multiple molecular studies have unravelled distinct (epi)-genetic subgroups of ‘histone wildtype’ pedHGG correlated with a variety of molecular and clinical features [19, 20, 26]. Recent studies on ‘HGG’ in infants have also shown that some of these tumors are biologically very different from those in older children, harbour recurrent, targetable gene fusions, and have a favourable prognosis despite histological features of malignancy [8]. Similarly, low-grade gliomas have also been found to be molecularly heterogeneous, with a number of subgroups showing consistent patterns of histological features, molecular profiles and genetic alterations (e.g. [10, 35, 36, 43, 57] and reviewed in [16]).

Interestingly, recent reports have identified a handful of pediatric brain tumors displaying a fusion of the *PATZ1* gene with either *MN1* or *EWSR1* as a partner [1, 4, 6, 14, 35, 38, 42, 45] (summarized in Table 1, top panel). The histological diagnoses of these tumors included glial, glioneuronal and ‘polyphenotypic’ morphologies, hinting at some potential similarities but also variation in appearance, which may have prevented previous recognition as a defined entity. *EWSR1*:*PATZ1* fusions have also been reported in a subset of sarcomas occurring across a wide range of ages, with a predilection for occurrence in the chest wall, showing substantial heterogeneity in their morphology and immunoprofile [3, 7, 27, 28, 33, 48, 52].

**Table 1:** Summary of *PATZ1*-fused tumors previously reported in the literature

Study	No. of cases	Fusion reported	Histopathologic features (ID in this series)
‘CNS’ studies			
Chadda et al. [6]	1	*MN1*:*PATZ1*	Astroblastoma
Rossi et al. [38]	1	*EWSR1*:*PATZ1*	Infantile Glioblastoma WHO Grade 4. Round monomorphous nuclei, cells with clear cytoplasm and oligodendroglia-like morphology, rich vascular network, microvascular proliferation, and microcysts
Lopez-Nunez et al. [23]	1	*EWSR1*:*PATZ1*	Areas of cells with clear cytoplasm admixed with sheets of monotonous, round to spindled cells. “Malignant, poorly differentiated”
Burel-Vandenbos et al. [4]	1	*MN1*:*PATZ1*	Malignant neuroepithelial tumor: hyperchromatic, polymorphous nuclei; clear cells, perivascular and stromal hyalinization, perivascular pseudorosettes, microcysts. (PATZ1-066)
Stichel et al. [45]	3	*EWSR1*:*PATZ1* *MN1*:*PATZ1*	Malignant neuroepithelial tumor with sarcomatous differentiation (PATZ1-014), glioneuronal tumor (PATZ-024), neuroepithelial neoplasia (PATZ-025)
Siegfried et al. [42]	1	*EWSR1*:*PATZ1*	Low-grade glioneuronal tumor. Olig2 and synaptophysin positive cells with pleomorphic nuclei, vascular hyalinzation, pleomorphic clear cells (PATZ1-021)*
Alvarez-Breckenridge et al. [1]	1	*EWSR1*:*PATZ1*	Glioneuronal tumor (no further details)
Johnson et al. [14]	1	*EWSR1*:*PATZ1*	Low-grade glioma (no further details)
Qaddoumi et al. [35]	1	*EWSR1*:*PATZ1*	BRAF^V600E^ negative ganglioglioma (no further details)
‘Sarcoma’ studies			
Tsuda et al. [48]	3	*EWSR1*:*PATZ1*	Round cell sarcomas
Pei et al. [33]	1	*EWSR1*:*PATZ1*	GFAP positive and CD99 negative spinal intradural extramedullary tumor, eventually described as glioneuronal tumor. Monotonous spindle cells; abundant vasculature
Michal et al. [28]	9	*EWSR1*:*PATZ1*	Spindle and round cell sarcomas
Bridge et al. [3]	11	*EWSR1*:*PATZ1*	Morphologically variable; mostly undifferentiated or small round cell sarcomas, includes 4 CNS tumors
Chougule et al. [7]	2	*EWSR1*:*PATZ1*	Spindle and round cell sarcomas
Watson et al. [52]	5	*EWSR1*:*PATZ1*	Round cell sarcomas
Mastrangelo et al. [27]	1	*EWSR1*:*PATZ1*	Small round cell tumor

*IHC* immunohistochemistry

*PATZ-019 was also reported in Siegfried et al. [42], however, RNA-seq was only done on PATZ-021, revealing the *EWSR1*:*PATZ1* rearrangement

Here, we describe a distinct molecular tumor type identified by investigation of a large cohort of DNA methylation and associated genomic profiling data. The morphological heterogeneity, marker expression and pathognomonic fusions of *PATZ1* with either *MN1* or *EWSR1* in this group lead us to suggest a name of ‘neuroepithelial tumor with *PATZ1* fusion’ (NET-PATZ1).

## Materials and methods

### Tumor tissue and clinical data

Our cohort comprises a total of 60 patients. All available clinical data are listed in Supplementary Table 1. Where not reported, patient sex was predicted using data from the DNA methylation array. Tumor samples and clinical data were provided by multiple international collaborating centers and collected at the Department of Neuropathology of the Heidelberg University Hospital (UKHD) and at the German Cancer Research Center (DKFZ, Heidelberg, Germany) with approval from the respective institutional review boards/ethics committees and informed consent from all patients or their guardians.

### DNA methylation profiling and copy number variation calling

DNA methylation profiles on a genome-wide level were obtained using the Illumina Infinium HumanMethylation450 (450 k) or HumanMethylationEPIC (EPIC) BeadChip arrays according to the manufacturer’s instructions (Illumina, San Diego, USA). Raw data were generated at the Genomics and Proteomics Core Facility of the DKFZ, at the Neuropathology Department of the UKHD and at respective international contributing institutes, using both fresh-frozen and formalin-fixed paraffin-embedded (FFPE) tissue samples. All computational analyses were performed using R (http://www.R-project.org, R Development Core Team). Using the ‘conumee’ R package in Bioconductor (https://bioconductor.org/packages/release/bioc/html/conumee.html), the output data from the DNA methylation arrays were also used to investigate copy number alterations, which were assessed by manual inspection and in detail using IGV [37, 47]. The framework for data processing and downstream output after obtaining raw data from the methylation panels has been widely used and published elsewhere [5]. Summary copy number plots were generated using an in-house R-script (https://github.com/dstichel/CNsummaryplots).

### Histopathology and immunohistochemistry

Confirmed histopathological diagnoses for the tumors included in this series were obtained through our international collaborators, as reviewed per the local pathologist and/or reference pathologists. Two experienced neuropathologists (F.S., A.Ko.) re-evaluated tumor haematoxylin–eosin (H&E) slides centrally available for *n* = 18 cases. Diagnostic criteria used were according to the 2016 WHO Classification of CNS tumors [24]. Features assessed included cell density, cytoplasm and nuclear pleomorphism, the number of mitotic figures per ten high-power fields (HPF; one HPF = 0.238 mm^2^), presence of microvascular proliferation and necrosis, and infiltrative nature. Immunohistochemical staining with Ki-67, GFAP, MAP2, NeuN, Olig2, Synaptophysin, S-100, CD34 and Vimentin was performed for a subset of tumors either in Heidelberg or at other contributing centers (*n* = 16). NG2 immunostaining was performed with NG2/CSPG4 (E3B3G) XP Rabbit monoclonal antibody (Cell Signaling, Danvers, Massachusetts, USA, 1:200).

### Whole exome and gene panel sequencing

For *n* = 4 cases, whole exome sequencing (WES) data were generated for the INFORM study, from tumor and matched blood DNA, as previously described [54]. Gene panel sequencing data were available for *n* = 10 cases, including 7 cases for which normal blood DNA was also sequenced. The gene panel includes 130 commonly mutated genes in CNS neoplasms and has been described previously [39].

### RNA sequencing analysis

RNA sequencing data (total *n* = 27) for the purpose of fusion detection was either generated by contributing sites (*n* = 8) or locally in Heidelberg. For samples sequenced in Heidelberg, sequencing was performed from FFPE (*n* = 8) on the NextSeq500 (Illumina) with 75 bp paired-end reads, at the Neuropathology Department of UKHD, as previously described [54]. For additional quantitative gene expression analysis, sequencing of high-quality RNA from frozen tissue (*n* = 11) was performed by the High Throughput Sequencing Unit of the Genomics and Proteomics Core Facility (GPCF) at the DKFZ using the Illumina HiSeq4000 or NovaSeq platform, with libraries prepared according to the manufacturer’s instructions using the TruSeq Stranded mRNA Library Prep Kit. Gene expression data analysis was performed using the online tool ‘R2: Genomics Analysis and Visualization Platform’ (https://hgserver2.amc.nl/cgi-bin/r2/main.cgi). Detection of gene fusions was performed using the Arriba analysis tool (https://github.com/suhrig/arriba).

### Drug screening

The KS-1 cell line, from a 45-year-old female patient diagnosed with glioblastoma, was purchased from the Japanese Collection of Research Bioresources (JCRB) Cell Bank. RNA sequencing was performed to confirm the presence of the *MN1*:*PATZ1* fusion. The cells were subjected to a drug screen at the Hopp Children’s Cancer Center, Heidelberg (KiTZ) Translational Drug Screening Unit (TDSU), with a library consisting of 74 widely used anti-cancer drugs. Cells were seeded at *n* = 750 per well in 384-well plates, pre-printed with drugs at 5 different concentrations, in duplicate per concentration. Cell Titer Glo (CTG2.0, Promega) viability readouts were performed on a FLUOstar OPTIMA plate reader (BMG Labtech) 72 h after cell seeding and quantified as residual metabolic activity. All cell viability readouts were normalized to the highest DMSO concentration used for the suspension of the respective drug. Validation experiments were carried out for five selected drugs. To that end, 1500 cells per well were seeded into a U-bottom 96-well plate. Drug concentrations used for validation experiments were selected individually per compound such that the IC_50_ could be confirmed and specified more precisely and the maximum plateau effect could be validated.

## Results

### DNA methylation profiling

**Fig. 1:**
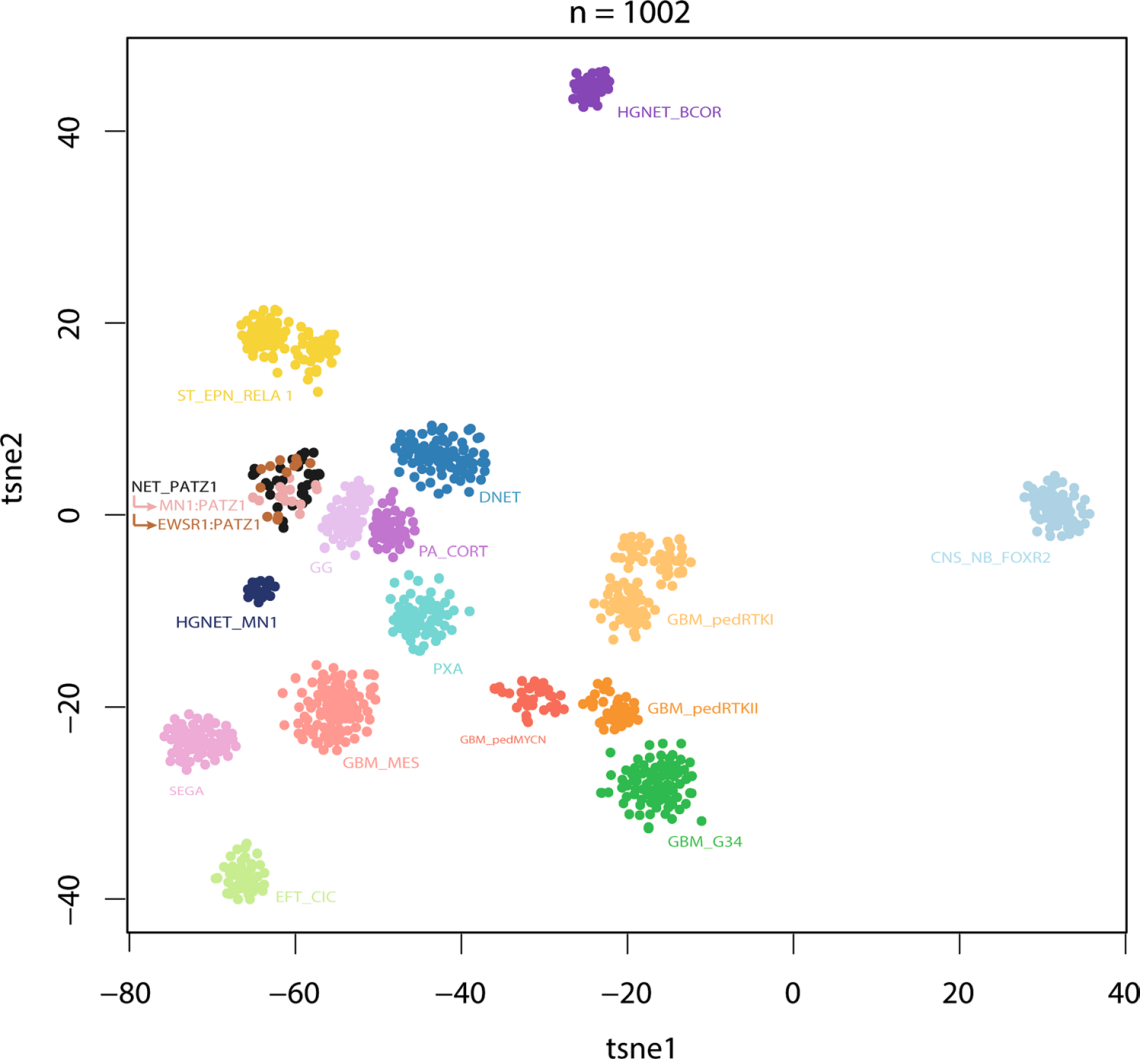
*t*-distributed stochastic neighbor embedding (tSNE) clustering of DNA methylation patterns of 60 NET-PATZ1 tumors alongside 942 in-house reference samples representing 15 other low- and high-grade glial and glioneuronal tumor types, using the 10,000 most variably methylated probes. NET-PATZ1 forms a distinct ‘island’. *CNS_NB_FOXR2* CNS neuroblastoma with FOXR2 activation; *DNET* dysembryoplastic neuroepithelial tumor; *EFT_CIC* CNS ewing sarcoma family tumor with CIC alteration; *GBM_G34* glioblastoma, H3.3 G34 mutant; *GBM_MES* glioblastoma, subclass mesenchymal; *GBM_pedMYCN* pediatric-type glioblastoma, subclass MYCN; *GBM_pedRTKI* pediatric-type glioblastoma, subclass RTKI; *GBM_pedRTKII* pediatric-type glioblastoma, subclass RTK II; *GG* ganglioglioma; *HGNET_BCOR* CNS high-grade neuroepithelial tumor with *BCOR* alteration; *HGNET_MN1* CNS high-grade neuroepithelial tumor with *MN1* alteration; *NET_PATZ1* neuroepithelial tumor with *PATZ1* fusion; *PA_CORT* hemispheric pilocytic astrocytoma; *PXA* pleomorphic xanthoastrocytoma; *SEGA* subependymal giant cell astrocytoma; *ST_EPN_RELA* supratentorial ependymoma, RELA fused

Routine diagnostic molecular profiling performed in Heidelberg in the context of the INFORM pipeline [54], the Molecular Neuropathology 2.0 or Pediatric Targeted Therapy 2.0 studies or otherwise in the Neuropathology Department of the UKHD [39, 45] revealed a small but recurring number of CNS tumors harboring fusions of the *PATZ1* gene coupled to either *MN1* or *EWSR1* (see below). When further investigating the DNA methylation profile of these tumors within the context of a much larger reference database, they were found to form a distinct molecular cluster with several other tumors from other sources. Visualization of the genome-wide methylation pattern using *t*-distributed stochastic neighbor embedding (tSNE; Supplementary Fig. 1, online resource) and uniform manifold approximation and projection (UMAP; not shown) confirmed a common pattern with broad proximity to other molecular types of both low- and high-grade glial and glioneuronal tumors. A selected analysis of the tumors in this novel cluster (*n* = 60) compared with the genome-wide DNA methylation profiles of a reference cohort consisting of 15 other low- and high-grade glial and glioneuronal tumor types, confirmed a clearly distinct grouping (Fig. 1). No similarity was seen with the recently described HGNET_MN1 tumor type, which is characterized by *MN1*:*BEND2* and *MN1*:*CXXC5* (but not *PATZ1*) fusions. When assessed by the current Heidelberg Brain Tumor Classifier, which uses a random forest-based class prediction algorithm based on the output of the methylation analysis (v11b6; https://www.molecularneuropathology.org/mnp), tumors of this cluster scored poorly for all currently known entities (calibrated scores < 0.6), thus supporting the novel and distinct nature of this molecular type. Given their varied morphological appearance and defining fusions of *PATZ1*, as outlined further below, we provisionally suggest the term ‘neuroepithelial tumor with *PATZ1* fusion’ to describe this molecular type.

### Next-generation DNA and RNA sequencing analysis

Sequencing data of tumor DNA only (*n* = 3) or matched tumor-normal DNA pairs (*n* = 11), available through different platforms (gene panel or WES) revealed generally ‘quiet’ tumors at the level of point mutations or small insertions/deletions. Considering known CNS tumor-relevant genes, no mutations in genes such as *IDH1/2*, *H3F3A*, *BRAF*, or *TP53* were detected. There were also no recurrent driver mutations found in the data, and no evidence for germline mutations in cancer predisposition genes in any of the cases with available germline sequencing data.

**Fig. 2:**
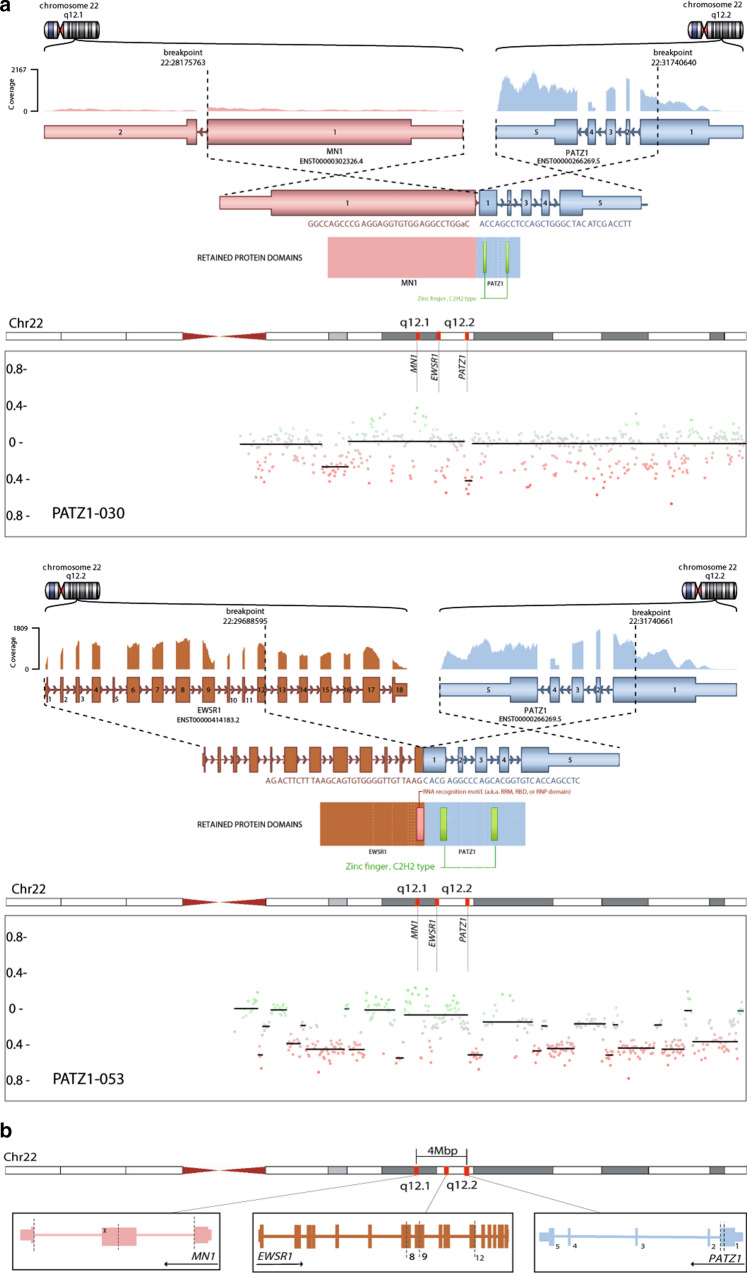
**a** Illustration of the *PATZ1* fusion genes detected by RNA-seq for two selected cases; and corresponding copy number plots of Chromosome 22. The three involved genes *MN1*, *EWSR1* and *PATZ1* are marked*.* PATZ1-030 harbors an in-frame *MN1*:*PATZ1* fusion, retaining an intronic pseudoexon (upper panel). PATZ1-053 demonstrates a variant fusion transcript juxtaposing Exon 12 of *EWSR1* onto the usual partner 3′ sequence of *PATZ1*, in contrast to the more prevalent 5′ breakpoints observed in Exons 8 and 9 of *EWSR1* (lower panel). **b** Schematic view of the loci of the genes involved in the fusions described (*MN1*, *EWSR1*, and* PATZ1*)*.* Note the proximity of the three genes (all lie within approximately 4 Mbp). The dashed lines resemble the genomic breakpoints observed in the gene fusions. An intronic breakpoint observed in a subset of *MN1_PATZ1* fusions introduces a novel pseudoexon (marked with x) whilst maintaining the reading frame. Isoforms illustrated: *EWSR1* NM_005243; *PATZ1* NM_0.13986.4; *MN1* NM_002430.3

In contrast, the results of the RNA sequencing analysis were more informative. Supplementary Table 1 describes the RNA-seq fusion detection output for the 27 tumors with data available. Strikingly, in all cases where RNA-seq was conducted (27/27, 100%), an in-frame fusion gene involving *PATZ1* was detected, with either *MN1* or *EWSR1* as the 5′ partner. This includes five of the aforementioned cases that have recently been previously published [4, 42, 45]. The two N′ terminal partners were roughly evenly distributed, with 13 *EWSR1*:*PATZ1* fusions (48%) and 14 *MN1*:*PATZ1* fusions identified (52%). The breakpoint within *PATZ1* is unusual, with most fusion junctions occurring in the middle of exon 1 rather than at a splice junction. The remaining exons 2–5 of *PATZ1* are also retained, conserving the zinc finger (C2H2 type) structure as part of the fusion product (Fig. 2). Most *MN1*:*PATZ1* fusions showed a break at the end of *MN1* exon 1. In some cases, however, an intronic breakpoint introduced an in-frame fusion with inclusion of an additional novel *MN1* exon. The *EWSR1*:*PATZ1* fusions harbor *EWSR1* exons 1–8, 1–9 or 1–12. These findings are in line with the breakpoints detected in the previously reported CNS and sarcoma *PATZ1* fusions [1, 14, 35, 52].

**Fig. 3:**
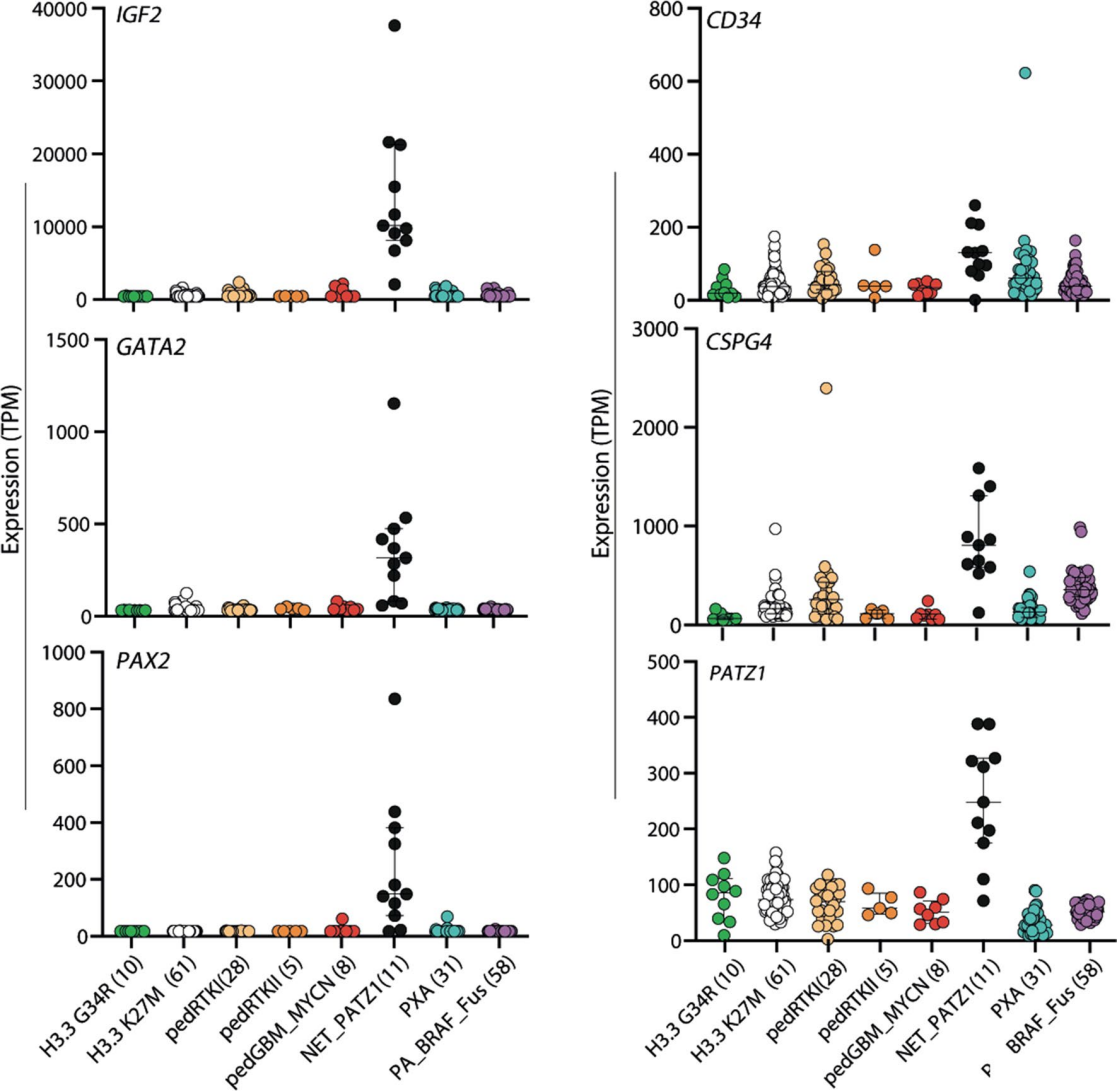
Differential expression analysis between NET-PATZ1 and a reference cohort of other glioma subtypes; *IGF2*, *GATA2*, *PAX2* and *PATZ1* and are more highly expressed in NET-PATZ1 cases when compared with representative pediatric HGG or LGG tumors, while CD34 and NG2 (*CSPG4*) could represent potential IHC staining markers for NET-PATZ1. Gene expression values are shown as TPM (transcripts per kilobase million). Where relevant, bars indicate median and 1st/3rd Quartile. *H3.3 G34R* glioblastoma, IDH wildtype, H3.3 G34 mutant; *H3.3 K27M* diffuse midline glioma H3 K27M mutant: *pedGBM_MYCN*, glioblastoma, IDH wildtype, subclass MYCN; *pedRTKI*, glioblastoma, IDH wildtype, subclass RTK I; *pedRTKII*, glioblastoma, IDH wildtype, subclass RTK II; *PXA* pleomorphic xanthoastrocytoma; *PA_BRAF_Fus* pilocytic astrocytoma with BRAF fusion

For RNA-seq data generated from high-quality frozen tumor tissue (*n* = 11), we also performed an exploratory differential gene expression analysis (with the caveat that the sample size is small). For this, we examined genes that were significantly differentially expressed between NET-PATZ1 and a combined reference cohort of other glioma subtypes (ANOVA test, *p* < 0.00001, corrected for multiple testing using FDR). This revealed a total of 964 upregulated transcripts in NET-PATZ1 compared with the reference samples, and 156 significantly down-regulated genes (Supplementary Table 2, online resource). The *PATZ1* gene itself is more highly expressed in these cases when compared with other representative pediatric HGG or LGG tumors (Fig. 3). Additional candidates of potential interest within the top upregulated genes included *IGF2*, *PAX2* and *GATA2*, with known roles in brain stem cell biology, differentiation and development [18, 49, 53, 58], although these data will need confirming in a larger series. Looking in a supervised way at expression of particular differentiation markers commonly used for immunohistochemical analysis of glial/glioneuronal tumors, we found low *GFAP* expression, and modest levels of *OLIG2*, NeuN (*RBFOX3*), *MAP2* and synaptophysin (*SYP*). Expression of *CD34* and *CSPG4* (NG2) is higher than in the other comparison groups, opening possible avenues for diagnostic IHC staining. *MKI67* levels as a surrogate for proliferation were higher than in pilocytic astrocytoma (PA), but lower than the GBM subgroups (Fig. 3, Supplementary Fig. 3, online resource). In contrast to reports on *EWSR1*:*PATZ1*-fused sarcoma [3, 7, 27, 52], there was no appreciable or distinguishing expression of *CD99* or *desmin* (Supplementary Table 2; Supplementary Fig. 3, online resource) or other muscular markers such as *MYOD1*, *myogenin*, *CALD1*, or *SOX10* in the present series (not shown).

### Copy number alterations of chromosome 22 are frequent in NET-PATZ1

**Fig. 4:**
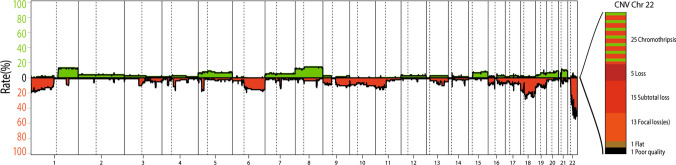
Summary plot of copy number alterations in NET-PATZ1 and categorization of copy number alterations observed on chromosome 22, the most frequent being a dramatic shattering pattern (chromothripsis)

A summary of the copy number alterations identified in the combined cohort is given in Fig. 4. At the global level, the tumors are relatively ‘quiet’, with few recurrently altered regions. We excluded one tumor from the CNV analysis due to poor data quality (remaining *n* = 59). Chromosomal arms 1p (10/59, 17%), 1q (10/59, 17%) and 6q (10/59, 17%) and chromosome 18 (16/59, 27%) were most frequently affected at the level of broad changes. In comparison with data regarding *PATZ1*-fused sarcoma, the *PATZ1*-fused CNS tumors in this series were not enriched for homozygous deletions mapping to the *CDKN2A/B* locus (observed in only 2/59 cases, 3%) [3]. The most notable feature, however, is the presence of recurrent structural copy number variations on chromosome 22, seen in 98% of the cases (58/59). Among those alterations, the most frequently observed event was a chromothripsis-like, shattered pattern along chromosome 22 (25/59, 42%). Supplementary Fig. 2, online resource provides a closer look at the copy number variations observed on chromosome 22. These structural alterations are presumably driving the fusion formation through an intra-chromosomal arrangement, since *EWSR1*, *MN1* and *PATZ1* are all located on chromosome 22 (Fig. 2b). This high frequency of copy number alterations, including in cases where no RNA data were available, further supports the suggestion that *PATZ1* fusion is likely a defining feature of this molecular tumor type.

### Clinical parameters and morphological aspects

**Fig. 5:**
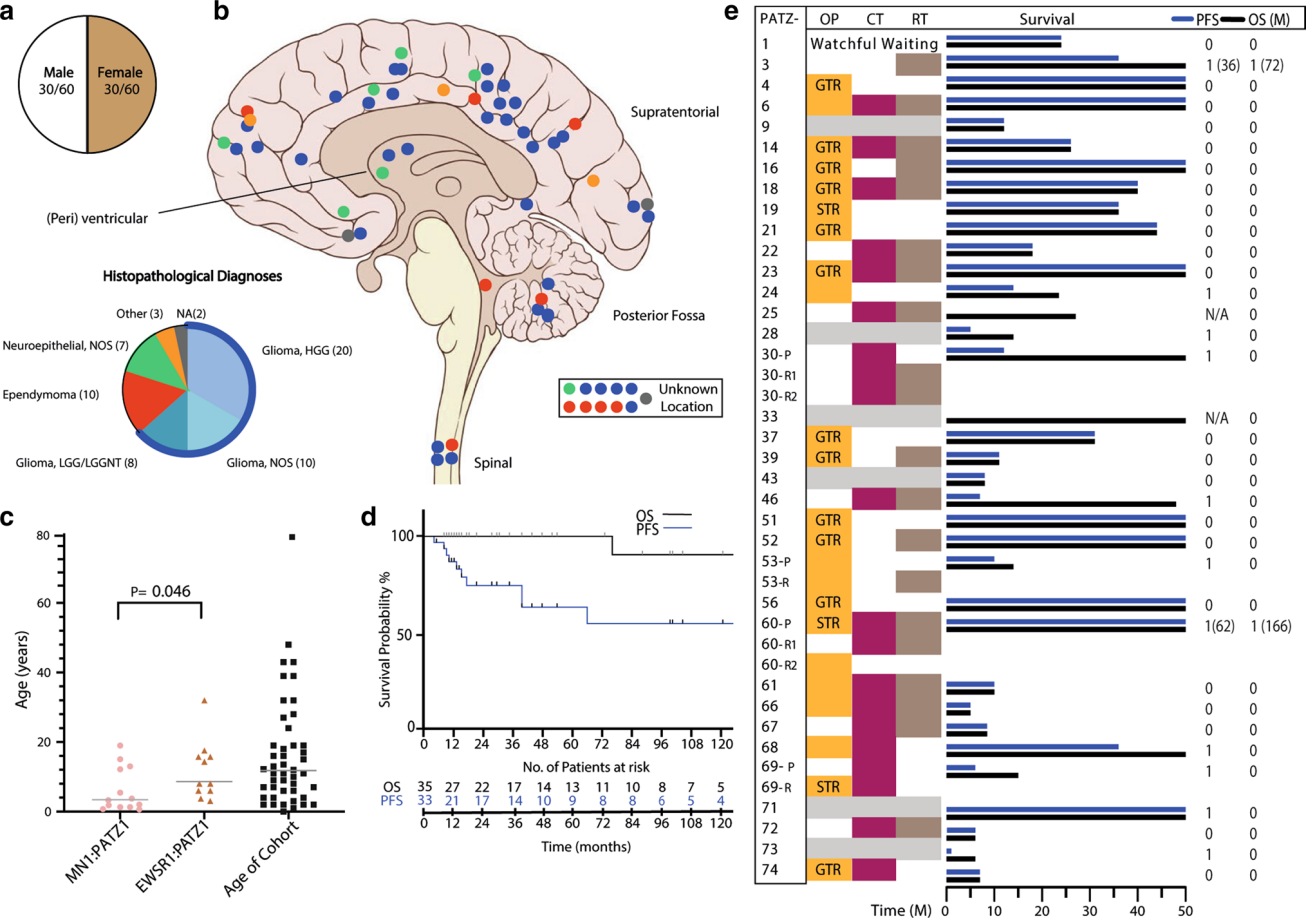
Clinical features of NET-PATZ1. **a** Patient sex distribution. **b** Distribution of tumor location. The institutional histopathological diagnoses of the series are also shown, representing a broad spectrum of mostly glial diagnoses. **c** Age distribution with the horizontal line representing median age of our cohort (11 years). MN1:PATZ1-fused tumors appear to be significantly enriched in younger ages (median = 3.5 years) vs EWSR1:PATZ1 (median = 8 years), (*p* value; 0.046, Student’s *t* test). **d** Clinical outcome in terms of OS and PFS of NET-PATZ1. **e** Overview of the different therapy protocols NET_PATZ1 patients within this cohort received. Where highlighted, the patients received that particular therapy modality, grey bars indicate unknown data. Different management as per primary vs relapse tumor is also shown. Note that some patients benefited from surgery (OP) alone. *CT* chemotherapy, *RT* radiation therapy, *P* primary, *R (1/2)* relapse 1 or relapse 2, *GTR* gross total resection, *STR* subtotal resection, *1* relapse/death, *0* censored. Detailed therapy protocols are listed in Supplementary Table 1, online resource

All clinical data available for our cohort are outlined in Fig. 5. More detailed descriptions are given in Supplementary Table 1, online resource. There is no sex-specific predominance observed in NET-PATZ1 (M:F = 1:1; Fig. 5a). Among the samples where the tumor location was known (*n* = 49), NET-PATZ1 most commonly showed a supratentorial manifestation (36/49 hemispheric, 4/49 peri- and intraventricular, total 40/49, 82%). Five of the 49 tumors (10%) were located in the posterior fossa, and 4/49 (8%) were spinal, including 2 metastatic lesions (Fig. 5b).

Figure 5c shows the age distribution for NET-PATZ1. The median age in our cohort was 11.0 years (range 0–80), with 74% of tumors occurring in patients under 18 years of age, indicating that NET-PATZ1 is primarily a childhood disease. Because some tumor samples were from recurrent tumors, and data about patient age could not always be confirmed to be the age at initial diagnosis, we analyzed the subgroup with definite age at primary diagnosis separately (*n* = 40), but this did not show any significant differences from the overall cohort (median age 9 years, *p* value; 0.42, Mann–Whitney *U* test). Interestingly, when looking deeper into cases with a confirmed fusion variant, *MN1*:*PATZ1*-fused tumors seem to manifest at a younger age (median = 3.5 years) vs *EWSR1*:*PATZ1*-fused tumors (median = 8 years; *p* value = 0.046, Student’s *t* test).

The original histopathological diagnoses of the cases described in this series were very diverse and variably polyphenotypic (Fig. 5b, Supplementary Table 1, online resource). The most common pathologic diagnoses described a high-grade astrocytic histology, including glioblastoma or anaplastic astrocytoma (high-grade glioma, HGG) in 20/58 annotated tumors (34%). Ten tumors (17%) had an institutional diagnosis of ependymal morphology, including two subependymomas. A further seven tumors (12%) had low-grade glial or glioneuronal diagnosis, and seven further tumors were broadly described as neuroepithelial. Across the morphological spectrum provided, five tumors were noted as additionally having a ‘sarcomatous’ or mesenchymal differentiation pattern on a glioneuronal/neuroepithelial background.

**Fig. 6:**
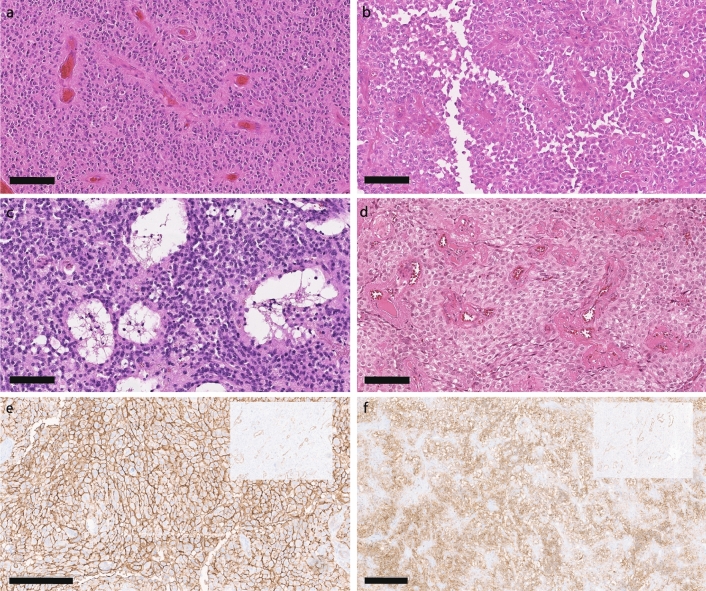
Histology of NET-PATZ1 tumors. **a** PATZ1-012: perivascular pseudorosettes resembling ependymal morphology are observed. **b** PATZ1-040 showed an astroblastoma-like morphology. **c** PATZ1-025: high cellularity was consistently observed across almost all cases. Microcysts are here encircled by monomorphous nuclei. **d** PATZ1-039: in this tumor monomorphous nuclei with clear cell morphology along with perivascular pseudorosettes were encountered. **e**, **f** NG2 staining in PATZ-030 and PATZ-014, respectively, with negative control insets (IDH WT GBM). Thick scale bars represent 100 µm, thin scale bars represent 200 µm

Given this very varied spectrum, we reassessed this novel tumor entity for any unifying histopathologic features. Figure 6 and supplementary Fig. 4, online resource show the extensively polyphenotypic morphology of NET-PATZ1. Histologically, hypercellularity was observed in almost all tumors (17/18, 94%). Nuclear morphology was sometimes, but not uniformly, irregular, with 8/18 tumors (57%) showing monomorphous nuclei with either large or small cells, arranged in clusters in two cases. Spindle-shaped cells were observed in 5/18 cases (28%). Mitoses were scarce for the centrally re-evaluated tumors, with only 1 tumor showing > 5 mitoses per 10 high-power fields (HPFs, one HPF = 0.238 mm^2^). However, most tumors (17/18, 94%) showed microvascular proliferation, with four tumor showing marked perivascular hyalinization. Necrosis was present in 33% of the cases (6/18), and was correlated with the presence of pseudorosettes and/or astroblastoma-like morphology. Details about our re-assessment are outlined in Table 2. A diffuse infiltration pattern was observed in 3/18 tumors (17%), with one tumor showing a cell cluster infiltration front. The clear biphasic and mesenchymal differentiation pattern of a subset of the tumors was also evident. However, within the five tumors showing sarcomatous or spindle cellular morphology, no correlation to a specific fusion partner (*MN1*/*EWSR1*) was observed.

**Table 2:** Summary of the re-evaluated histopathologic findings for tumor samples with available material

#	Detailed institutional histopathological diagnosis	Cell density	Cytoplasm and nuclei pleomorphism	Mitotic figures/10 HPF	Microvascular proliferation	Necrosis	Infiltration	Notes/fusion detected
4	Pleomorphic xanthoastrocytoma	High	Slightly pleomorphic, focally spindle cells	0	Hyalinized	No		*EWSR1*:*PATZ1*, perivascular pseudorosettes, biphasic differentiation
10	Glioblastoma WHO grade 4	High	Monomorphous nuclei, single giant cells	2	Few	No		Glial
12	Anaplastic ependymoma WHO grade 3	High	Monomorphous nuclei	1	Yes	Yes		Pseudorosettes
13	N/A	Moderate	Monomorphous, small, arranged in groups/lobules	0	Hyalinized	No		Microcysts, biphasic differentiation
14	Malignant NET with focal glial and sarcomatous differentiation	High	Small cell, partially spindle-shaped	0	No	No		*MN1*:*PATZ1*, biphasic differentiation
22	Giant cell glioblastoma WHO grade 4	High	Pleomorphic, small and large cells	0	Yes	No		*MN1*:*PATZ1*, biphasic differentiation
23	Glioblastoma WHO grade 4	High	Focally very pleomorphic, giant cells	3	Hyalinized	No	Diffuse	Focally spindle cells, biphasic differentiation
24	Low-grade glial/glioneuronal tumor	High	Pleomorphic, small and large cells	0	Yes	No	Diffuse	*EWSR1*:*PATZ1*
25	Malignant neuroepithelial tumor	High	Monomorphous nuclei	0	Yes	No		*EWSR1*:*PATZ1*, microcysts
28	Pleomorphic xanthoastrocytoma	High	Monomorphous, single giant cells, focally spindle cells	0	Few	No	Strong infiltration	*MN1*:*PATZ1*
30	High-grade glioma	High	Pleomorphic, focally spindle-shaped, many apoptotic bodies	8	Yes	Yes		*MN1*:*PATZ1*, focally perivascular pseudorosettes, biphasic differentiation
39	Anaplastic clear cell ependymoma WHO grade 2	High	Monomorphous, clear cells	0	Yes	No		Ependymal, pseudorosettes
40	Anaplastic ependymoma WHO grade 3	High	Monomorphous nuclei	0	Yes	Yes		*EWSR1*:*PATZ1*, astroblastoma-like
46	High-grade glioma	Moderate/high	Fibrillary, monomorphous nuclei	0	Yes	Yes	Diffuse	*MN1*:*PATZ1*, pseudorosettes, astroblastoma-like
54	Glioblastoma WHO grade 4	High	Monomorphous, small clusters of cells	1	Yes	No	Clusters	Infiltration in small clusters of cells
55	N/A	High	Moderate nuclear pleomorphism	1	Yes	Yes		*MN1*:*PATZ1*, pseudorosettes
56	Glioblastoma WHO grade 4; Gliosarcoma	High	Spindle-shaped cells	0	Yes	No		Sarcomatous differentiation
71	Glioblastoma WHO grade 4	High	Slightly pleomorphic, focally clear cells, prominent nucleoli	5	Hyalinized	Yes		*EWSR1*:*PATZ1*, perivascular pseudorosettes, focally spindle cells, biphasic differentiation

Details about immunohistochemistry is listed in Supplementary Table 1

*N/A* not applicable

Insufficient unstained sections were available to perform a comprehensive immunohistochemical analysis for most cases. The limited available data suggest variable staining for OLIG2 and GFAP, similar to the gene expression data, which also outlined the potential role of NG2 for future investigation (Fig. 6e, f). We exploited this finding and found specific positive staining in all cases prospectively evaluated where material was available (*n* = 4/4). Immunoprofiling data from previously reported PATZ1-fused CNS tumors included in this series are summarized in Supplementary Table 1, online resource.

Overall, given the absence of clear uniform histopathological or immunophenotypic features to classify the tumors as being of glial/ependymal versus neural origin, we provisionally propose the non-specific term ‘neuroepithelial tumor’ (NET) within the NET-PATZ1 nomenclature. We anticipate that in time, this unspecific term may be replaced by a clearer description if new evidence on cellular origins emerges.

### Patient outcomes may suggest an intermediate malignancy grade

Follow-up data were obtained for 35 patients, as listed in Supplementary Table 1, online resource, with a median follow-up period of 31 months (range 6–288 months). Eleven of the patients experienced a relapse during the follow-up period, including two patients with multiple relapses and spinal metastasis (PATZ-030, PATZ-068). Median PFS was 144 months. Only two of the patients died of their disease during the follow-up period, at 72 and 166 months after diagnosis (having relapsed at 36 and 62 months, respectively) (Fig. 5d). This clinical course is broadly compatible with the moderate *MKI67* (the gene encoding Ki-67) expression seen in the RNA data, and the mostly rare mitoses observed histologically. The limited follow-up data and the heterogeneity of treatments applied (Fig. 5e, Supplementary Table 1, online resource) preclude a definitive statement regarding the malignancy grade of this group of tumors, or a precise estimate of their overall survival rates or median OS. Our initial data, however, suggest an intermediate grade and notably better outcome than expected for true ‘high-grade’ tumors—important given that a third of cases initially received a diagnosis of HGG. Also, it is notable that a subset of patients maintained a stable disease despite being treated with surgery alone.

### Drug screening identifies potential candidates for future treatments

Screening of cell line molecular data in the Broad Institute’s Cancer Dependency Map portal (DepMap, https://depmap.org/portal/) revealed a CNS tumor cell line harboring an *MN1*:*PATZ1* fusion. This line, KS-1, is derived from a 45-year-old female patient diagnosed with a glioblastoma. The line grows both adherently or in neurosphere conditions, and confirmatory RNA sequencing of the line validated the presence of the fusion. Although this long-term in vitro culture certainly will not recapitulate all features of the primary tumors, we nevertheless performed an initial drug screening to obtain some possible first hints at future therapeutic options. The drug screen was performed using the Hopp Children’s Cancer Center, Heidelberg (KiTZ) Translational Drug Screening Unit (TDSU) library. This library comprises 74 approved cancer drugs targeting a variety of different pathways. The output chemiluminescence intensities were fed into the web-based BREEZE drug screen analysis pipeline (https://breeze.fimm.fi); [34]), which calculates a drug-specific sensitivity (DSS) score for each drug [56]. DSS scores were also compared to the chemostatic effect on normal fetal astrocytes, to assess tumor specificity. Data analysis yielded five drugs that emerged as potential candidates due to their superior efficacy in tumor cells: paclitaxel, d-actinomycin, volasertib (PLK1 inhibitor), navitoclax (BCL-2/BCL-XL/BCL-w inhibitor) and I-BET-151 (a Bromodomain family inhibitor) (Supplementary Fig. 5a, online resource). These drugs, particularly paclitaxel, D-actinomycin and volasertib, showed similar effects when subsequently validated individually in a small scale tissue culture setting (Supplementary Fig. 5b, online resource), and may thus represent a starting point for future preclinical studies in this tumor type.

## Discussion

Recent reports on a small number of pediatric brain tumors showing *PATZ1* fusions, as well as the detection of further such cases through our ongoing molecular diagnostic studies, prompted us to investigate the properties of these cases in more detail. In this study, we identified a distinct DNA methylation-based cluster of 60 tumor samples, within which all tumor samples where RNA sequencing was conducted (*n* = 27) were found to harbour a *PATZ1* fusion. We, therefore, believe it to be likely that these *MN1*:*PATZ1* and *EWSR1*:*PATZ1* fusions are pathognomonic for this novel tumor type.

### Diagnosis and origins of NET-PATZ1

Our findings suggest that when integrating morphological aspects and molecular analysis, unusual glial, ependymal or glioneuronal histology along with clustered, focal chr22 alterations may together give diagnostic hints for NET-PATZ1. As with the sarcomas in which *EWSR1*:*PATZ1* has been reported, the morphological variety, inconclusive immunoprofile and wide range of age at presentation are striking features that may account for the fact that these tumors have not been identified as a clear entity until now. When RNA sequencing cannot be performed to confirm presence of the fusion, methylation analysis and derived copy number data may be exploited as an alternative to establish the diagnosis of NET-PATZ1.

Exploring the differential diagnostic spectrum of this observation, the recently described HGNET-MN1 tumor type, which is characterized by alternative *MN1* fusions (mostly *MN1*:*BEND2* and *MN1*:*CXXC5*), manifests more frequently with an embryonal/astroblastoma histology and shows a clearly different DNA methylation profile [46]. The majority of NET-PATZ1 tumors presented with a histology extending along the glial or glioneuronal spectrum, but the scarce tissue available for immunohistochemistry together with marker gene expression analysis through RNA sequencing was inconclusive as to a clear lineage or distinct marker. Taken together with the mesenchymal phenotype noted in a subset of the cases in our cohort, we, therefore, advocate to provisionally call this tumor entity ‘neuroepithelial’ until a more concrete assessment of its likely origins can be made.

The observed overexpression of *IGF2*, *PAX2* and *GATA2* in NET-PATZ1 is also intriguing with respect to possible developmental origins and potential targets for therapeutic intervention. For example, *GATA2* has roles in fate determination of neural progenitors into midbrain GABAergic neurons [17], while *PAX2* is linked with development of the midbrain–hindbrain boundary and of GABAergic interneurons [11]. *IGF2* has multiple roles in brain development and disease, as reviewed for example in [2, 22]. *GATA2* has been reported to directly upregulate *IGF2* in chemo-resistant prostate cancer [51], while *IGF2* activity leads to *PAX2* overexpression in Wilms’ tumor [13], suggesting a possible signalling axis in NET-PATZ1. Of particular interest will be a future comparison with *PATZ1*-fused tumors occurring in non-CNS sites, to investigate the possibility of a common neuroectodermal/neural crest progenitor cell that may also be of relevance in the formation of the previously described sarcomas (although markers of *EWSR1*:*PATZ1* sarcomas such as CD99, desmin and myogenin were not found to be expressed in the CNS tumors). The histology and immunoprofile of *EWSR1*:*PATZ1*-fused sarcoma has also been described as incredibly polyphenotypic, but *MN1*:*PATZ1*-fused sarcomas have not yet been described.

An initial look at the survival data, with an average follow-up period of 31 months, indicates that NET-PATZ1 patients have a better overall survival than high-grade tumors (median OS not reached), despite experiencing several relapses (median PFS 144 months). This should prompt questioning of the nature of the GBM diagnoses in a subset of the patients included and could explain the observed survival pattern. This finding has consequences related to the aggressiveness and nature of the future therapy that is suitable for patients with tumors driven by this biology. However, more information is obviously needed to fully assess the malignancy of this tumor entity, particularly in the context of a uniform treatment protocol. Future retro- and prospective studies will, therefore, be critical for determining optimal clinical management.

### Potential oncogenic mechanisms and the role of PATZ1

*PATZ1* (POZ/BTB and AT Hook Containing Zinc Finger 1) is a transcription factor belonging to the POZ/BTB (Pox virus and Zinc finger/Broad-complex, tramtrack, and bric-à-brac) family and containing an AT-hook zinc finger domain [44]. It has been described as an important node as part of a network of transcription factors that maintain the “stemness” of embryonic stem cells [30], and a regulator of cellular reprogramming by inhibiting Pou5f1, depending on its expression levels. It has thus been assigned as both an activator and repressor of transcription, depending on the cellular context [25, 50].

*MN1* and *EWSR1* are both involved in fusions in other tumor entities. As described above, *MN1* alteration is one of the molecular hallmarks of the recently described HGNET-MN1 tumor subentity, while *EWSR1* has long been known to be involved in Ewing sarcoma, even though the exact factors underlying its oncogenicity remain to be fully understood [21]. *EWSR1*:*PATZ1* and similar oncogenic *EWSR1* fusions have been shown to cause globally altered transcriptional signatures [21, 52], and other *EWSR1* fusions have been detected in primary intra-axial tumors [23]. Both *MN1* and *EWSR1* have transcriptional activator activity [12, 29].

In the fusion described here, the transactivating domain of MN1/EWSR1 is retained, and fused to the zinc finger domain of PATZ1. It, therefore, seems likely that the downstream consequences of the fusion are determined by the aberrant recruitment of a transactivating domain at binding sites determined by the PATZ1 binding domain, perhaps together with upregulated expression via the *MN1*/*EWSR1* promoter, as seen, for example, with *EWSR1*:*FLI1* fusions in Ewing sarcoma [9, 12]. The exact details of the oncogenic mechanisms of *MN1*:*PATZ1* and *EWSR1*:*PATZ1*, however, are yet to be fully established and understood. Further studies will be needed to reveal the exact role of the fusion partners and the subsequent downstream effects in terms of global transcriptional deregulation.

## Summary

In conclusion, we describe here ‘neuroepithelial tumor with *PATZ1* fusion’ (NET-PATZ1)—a novel, molecularly distinct CNS tumor type with strikingly variable histopathologic morphology and heterogeneous multiphasic differentiation patterns. Being the sole molecular finding constant across all tumors analyzed, we postulate that the *PATZ1* fusions are a key driver of tumor initiation. Preliminary indications suggest an intermediate prognosis, although further studies will be needed to confirm this and to investigate the detailed biology, cellular origins, treatment sensitivity and clinical course of these tumors. The recognition of NET-PATZ1 as a defined tumor type, however, is an important step towards conducting such analyses, and will hopefully provide a foundation for optimized clinical management in future.

## Supplementary Information

Below is the link to the electronic supplementary material.

Supplementary file1(TIF 7263 kb)

Supplementary file2 (XLSX 78 kb)

Supplementary file3. Supplementary Fig. 1 *t*-distributed stochastic neighbor embedding (tSNE) visualization of DNA methylation patterns for our in house cohort including more than 80,000 bulk tumor samples, the platform on which mnp is based (https://www.molecularneuropathology.org/mnp). NET_PATZ1 cluster close to a variety of glial tumors but form a distinct ‘island’, representing a distinct molecular tumor type (TIF 27774 kb)

Supplementary file4. Supplementary Fig. 2 Copy number status observed on chromosome 22 for the two selected cases in Fig. 2. Top panel: B-Allele frequency (BAF) in the tumor at SNP positions which are heterozygous in the germline. 2nd, 3rd and bottom panel: Rescaled tumor: germline coverage ratio, indicating copy-number gains or losses; tumor and germline coverage. MN1, EWSR1 and PATZ1 loci are indicated (TIF 26046 kb)

Supplementary file5. Supplementary Fig. 3 Expression of particular differentiation/proliferation markers: *GFAP*, *MAP2*, *CD99*, Desmin (*DES*) *NeuN* (*RBFOX3*): low, *OLIG2* and Synaptophysin (*SYP*): modest. *MKI67* levels were higher than in PA, but lower than the GBM subgroups (similar to PXA). Gene expression is shown as TPM (transcripts per kilobase million). *H3.3 G34R*, glioblastoma, IDH wildtype, H3.3 G34 mutant; *H3.3 K27M*, diffuse midline glioma H3 K27M mutant: *pedGBM_MYCN*, glioblastoma, IDH wildtype, subclass MYCN; *pedRTKI*, glioblastoma, IDH wildtype, subclass RTK I; *pedRTKII*, glioblastoma, IDH wildtype, subclass RTK II; *PXA* pleomorphic xanthoastrocytoma; *PA_BRAF_Fus* pilocytic astrocytoma with BRAF fusion (TIF 14413 kb)

Supplementary file6. Supplementary Fig. 4 **a** PATZ1-013: marked perivascular hyalinization and microcysts are seen together with monomorphous nuclei and small cells arranged in lobules. **b** PATZ1-056 displayed spindle-shaped cells with strong resemblance to a mesenchymal phenotype. **c** Immunohistochemistry of available sections for PATZ1-056 revealed very sparse staining with antibodies against GFAP and negative staining for Olig2, synaptophysin and MAP2, in keeping with the gene expression-based analysis. Scale bars represent 100 µm. Supplementary Table 1 includes more information about the staining patterns seen in NET_PATZ1 (TIF 39143 kb)

Supplementary file7. Supplementary Fig. 5 **a** Heatmap of the primary drug screen conducted on the KS-1 cell line. Drugs are ordered according to the drug specific sensitivity score (DSS) of the KS-1 cells. Area under the curve for drug efficacy (AUC) and IC50 values are also shown. Five potentially interesting candidate hits were selected based on high DSS and superior efficacy in KS-1 compared with astrocytes. **b** The five candidate hits were tested again on the KS-1 cells with a greater range of concentrations to confirm efficacy and obtain a more precise IC50 value. X indicates that the IC50 value measured for fetal astrocytes is higher than the highest concentration of drug applied (drug cytotoxic effect was not completely reached)(XLSX 9176 kb)

## References

[1] Alvarez-Breckenridge C, Miller JJ, Nayyar N, Gill CM, Kaneb A, D’Andrea M (2017). Clinical and radiographic response following targeting of BCAN-NTRK1 fusion in glioneuronal tumor. NPJ Precis Oncol.

[2] Benarroch EE (2012). Insulin-like growth factors in the brain and their potential clinical implications. Neurology.

[3] Bridge JA, Sumegi J, Druta M, Bui MM, Henderson-Jackson E, Linos K (2019). Clinical, pathological, and genomic features of EWSR1-PATZ1 fusion sarcoma. Mod Pathol.

[4] Burel-Vandenbos F, Pierron G, Thomas C, Reynaud S, Gregoire V, Duhil de Benaze G (2020). A polyphenotypic malignant paediatric brain tumour presenting a MN1-PATZ1 fusion, no epigenetic similarities with CNS High-Grade Neuroepithelial Tumour with MN1 Alteration (CNS HGNET-MN1) and related to PATZ1-fused sarcomas. Neuropathol Appl Neurobiol.

[5] Capper D, Jones DTW, Sill M, Hovestadt V, Schrimpf D, Sturm D (2018). DNA methylation-based classification of central nervous system tumours. Nature.

[6] Chadda KR, Holland K, Scoffings D, Dean A, Pickles JC, Behjati S (2021). A rare case of paediatric astroblastoma with concomitant MN1-GTSE1 and EWSR1-PATZ1 gene fusions altering management. Neuropathol Appl Neurobiol.

[7] Chougule A, Taylor MS, Nardi V, Chebib I, Cote GM, Choy E (2019). Spindle and round cell sarcoma with EWSR1-PATZ1 gene fusion: a sarcoma with polyphenotypic differentiation. Am J Surg Pathol.

[8] Clarke M, Mackay A, Ismer B, Pickles JC, Tatevossian RG, Newman S (2020). Infant high-grade gliomas comprise multiple subgroups characterized by novel targetable gene fusions and favorable outcomes. Cancer Discov.

[9] Delattre O, Zucman J, Plougastel B, Desmaze C, Melot T, Peter M (1992). Gene fusion with an ETS DNA-binding domain caused by chromosome translocation in human tumours. Nature.

[10] Deng MY, Sill M, Chiang J, Schittenhelm J, Ebinger M, Schuhmann MU (2018). Molecularly defined diffuse leptomeningeal glioneuronal tumor (DLGNT) comprises two subgroups with distinct clinical and genetic features. Acta Neuropathol.

[11] Goode DK, Elgar G (2009). The PAX258 gene subfamily: a comparative perspective. Dev Dyn.

[12] Grünewald TGP, Cidre-Aranaz F, Surdez D, Tomazou EM, de Álava E, Kovar H (2018). Ewing sarcoma. Nat Rev Dis Primers.

[13] Hu Q, Gao F, Tian W, Ruteshouser EC, Wang Y, Lazar A (2011). Wt1 ablation and Igf2 upregulation in mice result in Wilms tumors with elevated ERK1/2 phosphorylation. J Clin Investig.

[14] Johnson A, Severson E, Gay L, Vergilio JA, Elvin J, Suh J (2017). Comprehensive genomic profiling of 282 pediatric low- and high-grade gliomas reveals genomic drivers, tumor mutational burden, and hypermutation signatures. Oncologist.

[15] Jones C, Baker SJ (2014). Unique genetic and epigenetic mechanisms driving paediatric diffuse high-grade glioma. Nat Rev Cancer.

[16] Jones DTW, Bandopadhayay P, Jabado N (2019). The power of human cancer genetics as revealed by low-grade gliomas. Annu Rev Genet.

[17] Kala K, Haugas M, Lillevali K, Guimera J, Wurst W, Salminen M (2009). Gata2 is a tissue-specific post-mitotic selector gene for midbrain GABAergic neurons. Development.

[18] Kala K, Haugas M, Lilleväli K, Guimera J, Wurst W, Salminen M (2009). *Gata2* is a tissue-specific post-mitotic selector gene for midbrain GABAergic neurons. Development.

[19] Korshunov A, Ryzhova M, Hovestadt V, Bender S, Sturm D, Capper D (2015). Integrated analysis of pediatric glioblastoma reveals a subset of biologically favorable tumors with associated molecular prognostic markers. Acta Neuropathol.

[20] Korshunov A, Schrimpf D, Ryzhova M, Sturm D, Chavez L, Hovestadt V (2017). H3-/IDH-wild type pediatric glioblastoma is comprised of molecularly and prognostically distinct subtypes with associated oncogenic drivers. Acta Neuropathol.

[21] Kovar H, Amatruda J, Brunet E, Burdach S, Cidre-Aranaz F, de Alava E (2016). The second European interdisciplinary Ewing sarcoma research summit–a joint effort to deconstructing the multiple layers of a complex disease. Oncotarget.

[22] Liu J, Speder P, Brand AH (2014). Control of brain development and homeostasis by local and systemic insulin signalling. Diabetes Obes Metab.

[23] Lopez-Nunez O, Cafferata B, Santi M, Ranganathan S, Pearce TM, Kulich SM (2020). The spectrum of rare central nervous system (CNS) tumors with EWSR1-non-ETS fusions: experience from three pediatric institutions with review of the literature. Brain Pathol.

[24] Louis DN, Perry A, Reifenberger G, von Deimling A, Figarella-Branger D, Cavenee WK (2016). The 2016 World Health Organization classification of tumors of the central nervous system: a summary. Acta Neuropathol.

[25] Ma H, Ow JR, Tan BCP, Goh Z, Feng B, Loh YH (2014). The dosage of Patz1 modulates reprogramming process. Sci Rep.

[26] Mackay A, Burford A, Carvalho D, Izquierdo E, Fazal-Salom J, Taylor KR (2017). Integrated molecular meta-analysis of 1,000 pediatric high-grade and diffuse intrinsic pontine glioma. Cancer Cell.

[27] Mastrangelo T, Modena P, Tornielli S, Bullrich F, Testi MA, Mezzelani A (2000). A novel zinc finger gene is fused to EWS in small round cell tumor. Oncogene.

[28] Michal M, Rubin BP, Agaimy A, Kosemehmetoglu K, Rudzinski ER, Linos K (2020). EWSR1-PATZ1-rearranged sarcoma: a report of nine cases of spindle and round cell neoplasms with predilection for thoracoabdominal soft tissues and frequent expression of neural and skeletal muscle markers. Mod Pathol.

[29] Miyake N, Takahashi H, Nakamura K, Isidor B, Hiraki Y, Koshimizu E (2020). Gain-of-function MN1 truncation variants cause a recognizable syndrome with craniofacial and brain abnormalities. Am J Hum Genet.

[30] Ow JR, Ma H, Jean A, Goh Z, Lee YH, Chong YM (2014). Patz1 regulates embryonic stem cell identity. Stem Cells Dev.

[31] Pajtler KW, Witt H, Sill M, Jones DT, Hovestadt V, Kratochwil F (2015). Molecular classification of ependymal tumors across all CNS compartments, histopathological grades, and age groups. Cancer Cell.

[32] Paugh BS, Zhu X, Qu C, Endersby R, Diaz AK, Zhang J (2013). Novel oncogenic PDGFRA mutations in pediatric high-grade gliomas. Cancer Res.

[33] Pei J, Zhao X, Patchefsky AS, Flieder DB, Talarchek JN, Testa JR (2019). Clinical application of RNA sequencing in sarcoma diagnosis: an institutional experience. Medicine.

[34] Potdar S, Ianevski A, Mpindi JP, Bychkov D, Fiere C, Ianevski P (2020). Breeze: an integrated quality control and data analysis application for high-throughput drug screening. Bioinformatics.

[35] Qaddoumi I, Orisme W, Wen J, Santiago T, Gupta K, Dalton JD (2016). Genetic alterations in uncommon low-grade neuroepithelial tumors: BRAF, FGFR1, and MYB mutations occur at high frequency and align with morphology. Acta Neuropathol.

[36] Ramkissoon LA, Horowitz PM, Craig JM, Ramkissoon SH, Rich BE, Schumacher SE (2013). Genomic analysis of diffuse pediatric low-grade gliomas identifies recurrent oncogenic truncating rearrangements in the transcription factor MYBL1. Proc Natl Acad Sci USA.

[37] Robinson JT, Thorvaldsdottir H, Winckler W, Guttman M, Lander ES, Getz G (2011). Integrative genomics viewer. Nat Biotechnol.

[38] Rossi S, Barresi S, Giovannoni I, Alesi V, Ciolfi A, Stefania Colafati G (2020). Expanding the spectrum of EWSR1-PATZ1 rearranged CNS tumors: an infantile case with leptomeningeal dissemination. Brain Pathol.

[39] Sahm F, Schrimpf D, Jones DT, Meyer J, Kratz A, Reuss D (2016). Next-generation sequencing in routine brain tumor diagnostics enables an integrated diagnosis and identifies actionable targets. Acta Neuropathol.

[40] Sahm F, Schrimpf D, Stichel D, Jones DTW, Hielscher T, Schefzyk S (2017). DNA methylation-based classification and grading system for meningioma: a multicentre, retrospective analysis. Lancet Oncol.

[41] Schwartzentruber J, Korshunov A, Liu XY, Jones DT, Pfaff E, Jacob K (2012). Driver mutations in histone H3.3 and chromatin remodelling genes in paediatric glioblastoma. Nature.

[42] Siegfried A, Rousseau A, Maurage CA, Pericart S, Nicaise Y, Escudie F (2019). EWSR1-PATZ1 gene fusion may define a new glioneuronal tumor entity. Brain Pathol.

[43] Sievers P, Appay R, Schrimpf D, Stichel D, Reuss DE, Wefers AK (2019). Rosette-forming glioneuronal tumors share a distinct DNA methylation profile and mutations in FGFR1, with recurrent co-mutation of PIK3CA and NF1. Acta Neuropathol.

[44] Siggs O, Beutler B (2012). The BTB-ZF transcription factors. Cell Cycle.

[45] Stichel D, Schrimpf D, Casalini B, Meyer J, Wefers AK, Sievers P (2019). Routine RNA sequencing of formalin-fixed paraffin-embedded specimens in neuropathology diagnostics identifies diagnostically and therapeutically relevant gene fusions. Acta Neuropathol.

[46] Sturm D, Orr BA, Toprak UH, Hovestadt V, Jones DTW, Capper D (2016). New brain tumor entities emerge from molecular classification of CNS-PNETs. Cell.

[47] Thorvaldsdottir H, Robinson JT, Mesirov JP (2013). Integrative genomics viewer (IGV): high-performance genomics data visualization and exploration. Brief Bioinform.

[48] Tsuda Y, Zhang L, Meyers P, Tap WD, Healey JH, Antonescu CR (2020). The clinical heterogeneity of round cell sarcomas with EWSR1/FUS gene fusions: impact of gene fusion type on clinical features and outcome. Genes Chromosomes Cancer.

[49] Urbánek P, Fetka I, Meisler MH, Busslinger M (1997). Cooperation of Pax2 and Pax5 in midbrain and cerebellum development. Proc Natl Acad Sci USA.

[50] Valentino T, Palmieri D, Vitiello M, Pierantoni GM, Fusco A, Fedele M (2013). PATZ1 interacts with p53 and regulates expression of p53-target genes enhancing apoptosis or cell survival based on the cellular context. Cell Death Dis.

[51] Vidal SJ, Rodriguez-Bravo V, Quinn SA, Rodriguez-Barrueco R, Lujambio A, Williams E (2015). A targetable GATA2-IGF2 axis confers aggressiveness in lethal prostate cancer. Cancer Cell.

[52] Watson S, Perrin V, Guillemot D, Reynaud S, Coindre JM, Karanian M (2018). Transcriptomic definition of molecular subgroups of small round cell sarcomas. J Pathol.

[53] Willett RT, Greene LA (2011). Gata2 is required for migration and differentiation of retinorecipient neurons in the superior colliculus. J Neurosci.

[54] Worst BC, van Tilburg CM, Balasubramanian GP, Fiesel P, Witt R, Freitag A (2016). Next-generation personalised medicine for high-risk paediatric cancer patients - the INFORM pilot study. Eur J Cancer.

[55] Wu G, Broniscer A, McEachron TA, Lu C, Paugh BS, Becksfort J (2012). Somatic histone H3 alterations in pediatric diffuse intrinsic pontine gliomas and non-brainstem glioblastomas. Nat Genet.

[56] Yadav B, Pemovska T, Szwajda A, Kulesskiy E, Kontro M, Karjalainen R (2014). Quantitative scoring of differential drug sensitivity for individually optimized anticancer therapies. Sci Rep.

[57] Zhang J, Wu G, Miller CP, Tatevossian RG, Dalton JD, Tang B (2013). Whole-genome sequencing identifies genetic alterations in pediatric low-grade gliomas. Nat Genet.

[58] Ziegler AN, Feng Q, Chidambaram S, Testai JM, Kumari E, Rothbard DE (2019). Insulin-like Growth Factor II: an essential adult stem cell niche constituent in brain and intestine. Stem Cell Rep.

