# Supplementary figures and images for: PATZ1 fusions define a novel molecularly distinct neuroepithelial tumor entity with a broad histological spectrum
Source: Acta Neuropathol. 2021 Aug 21;142(5):841–57. doi: 10.1007/s00401-021-02354-8 (PMC8500868; doi:10.1007/s00401-021-02354-8)

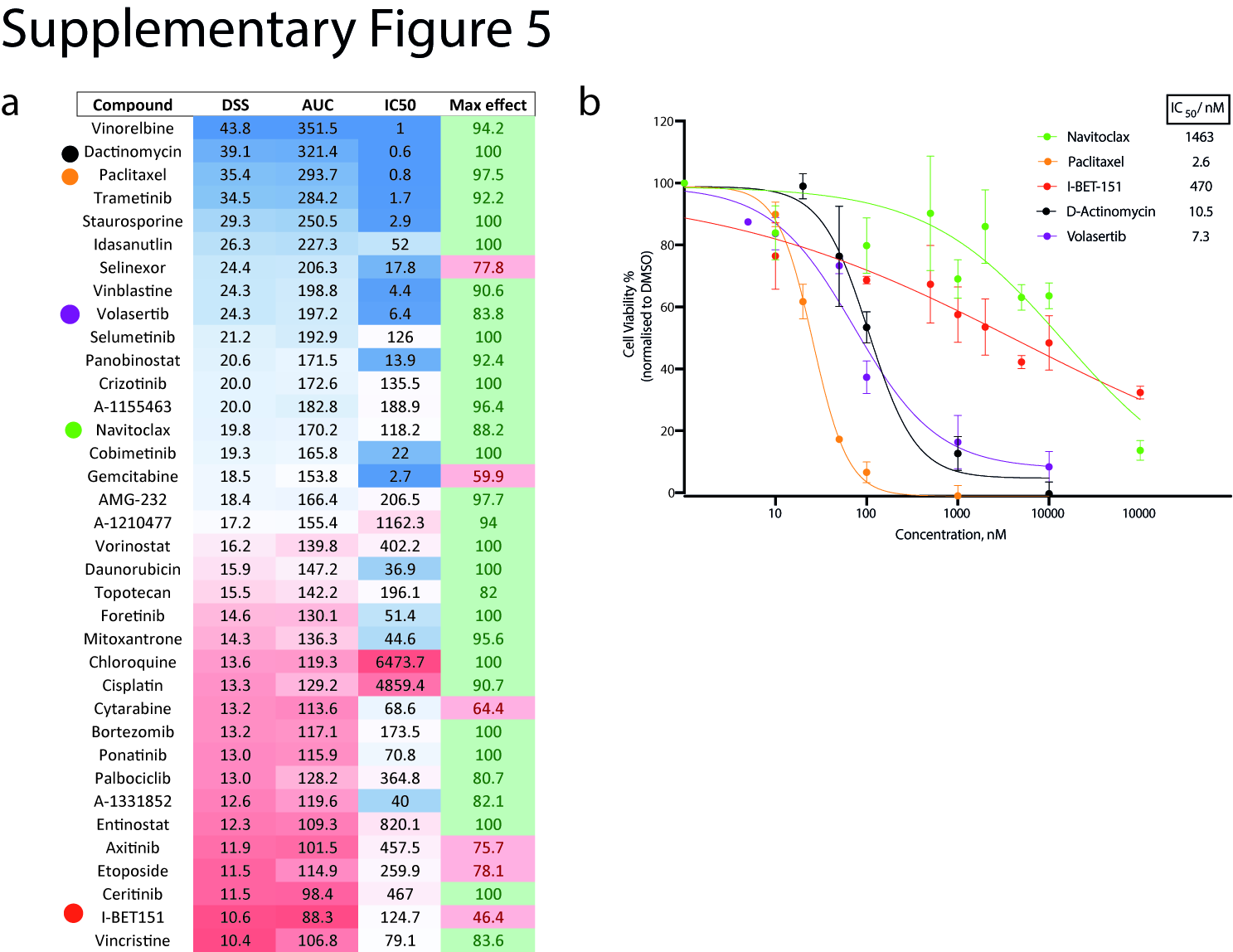

Supplement: Supplementary file 1 — Supplementary file1(TIF 7263 kb) [file 401_2021_2354_MOESM1_ESM.tif]

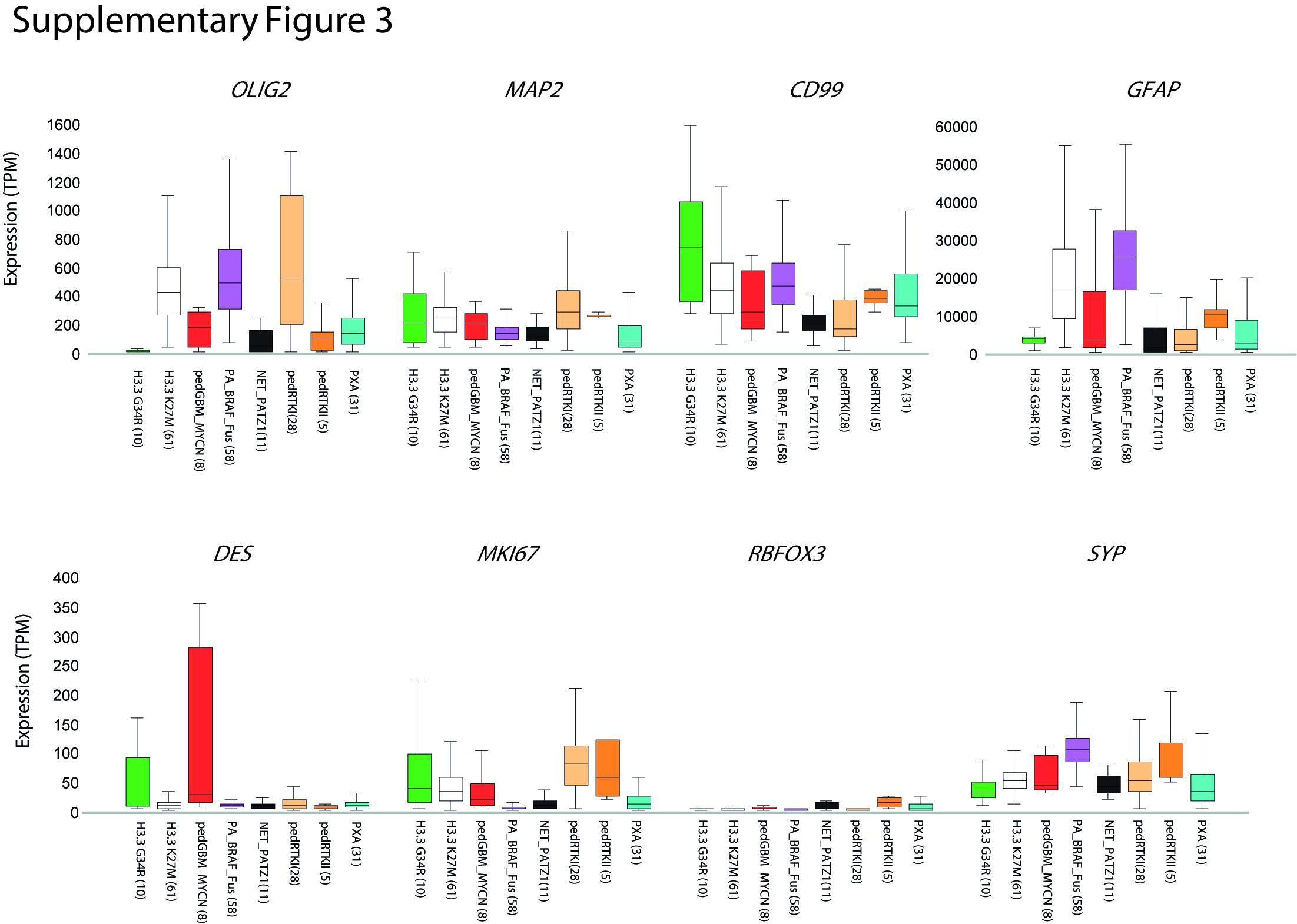

Supplement: Supplementary file 2 — Supplementary file2 (XLSX 78 kb) [file 401_2021_2354_MOESM2_ESM.tif]

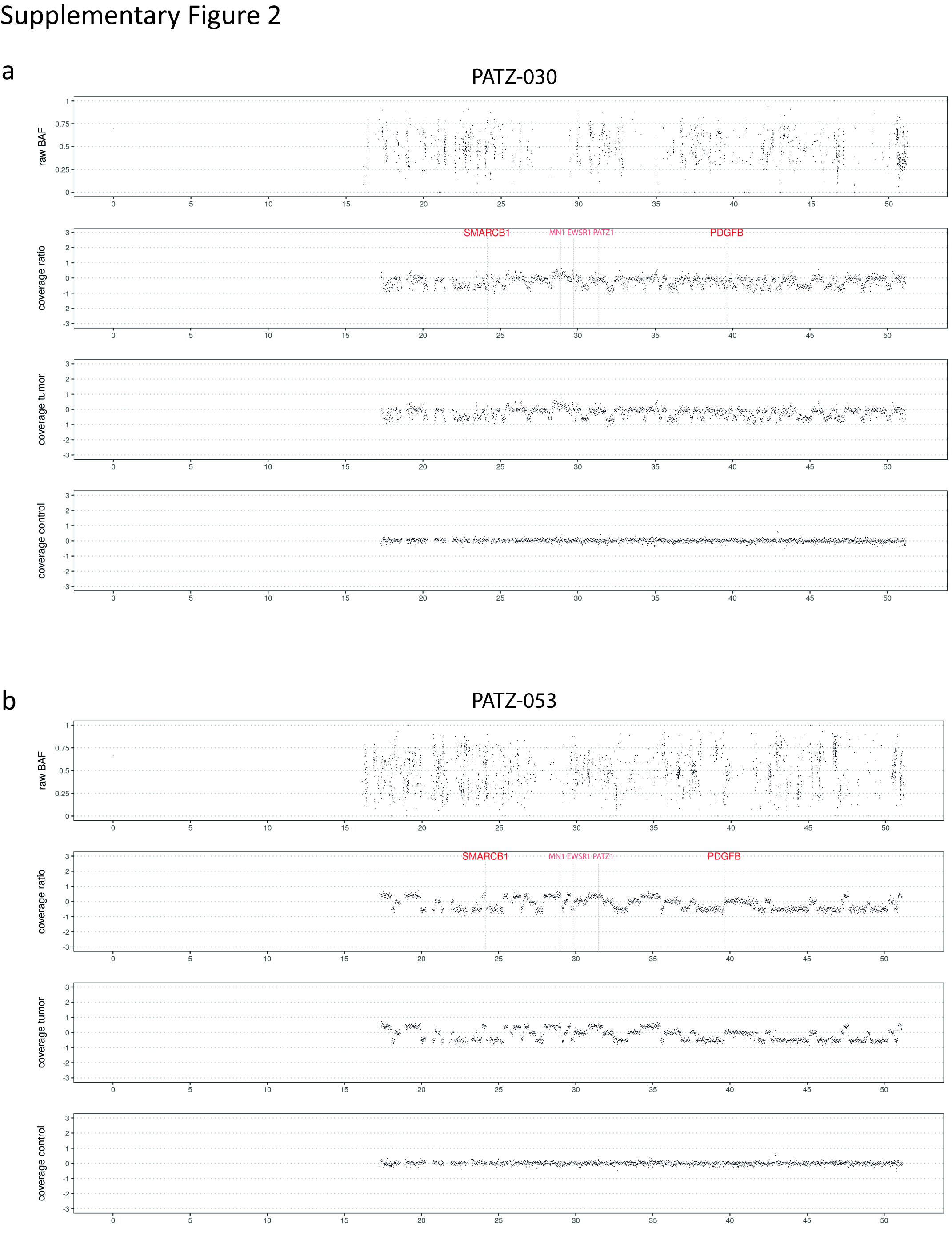

Supplement: Supplementary file 3 — Supplementary file3. Supplementary Fig. 1 t-distributed stochastic neighbor embedding (tSNE) visualization of DNA methylation patterns for our in house cohort including more than 80,000 bulk tumor samples, the platform on which mnp is based (https://www.molecularneuropathology.org/mnp). NET_PATZ1 cluster close to a variety of glial tumors but form a distinct ‘island’, representing a distinct molecular tumor type (TIF 27774 kb) [file 401_2021_2354_MOESM3_ESM.tif]

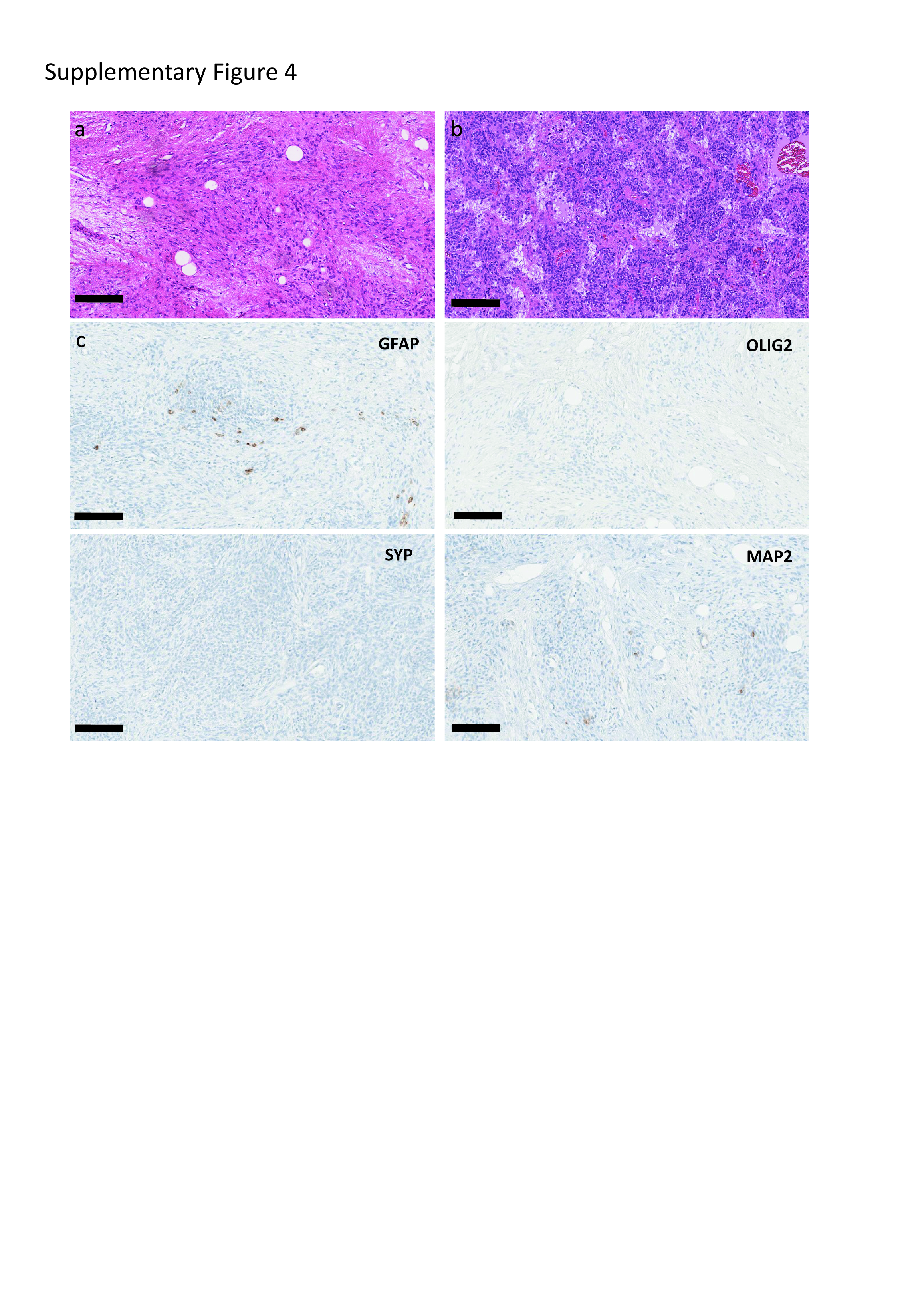

Supplement: Supplementary file 4 — Supplementary file4. Supplementary Fig. 2 Copy number status observed on chromosome 22 for the two selected cases in Fig. 2. Top panel: B-Allele frequency (BAF) in the tumor at SNP positions which are heterozygous in the germline. 2nd, 3rd and bottom panel: Rescaled tumor: germline coverage ratio, indicating copy-number gains or losses; tumor and germline coverage. MN1, EWSR1 and PATZ1 loci are indicated (TIF 26046 kb) [file 401_2021_2354_MOESM4_ESM.tif]

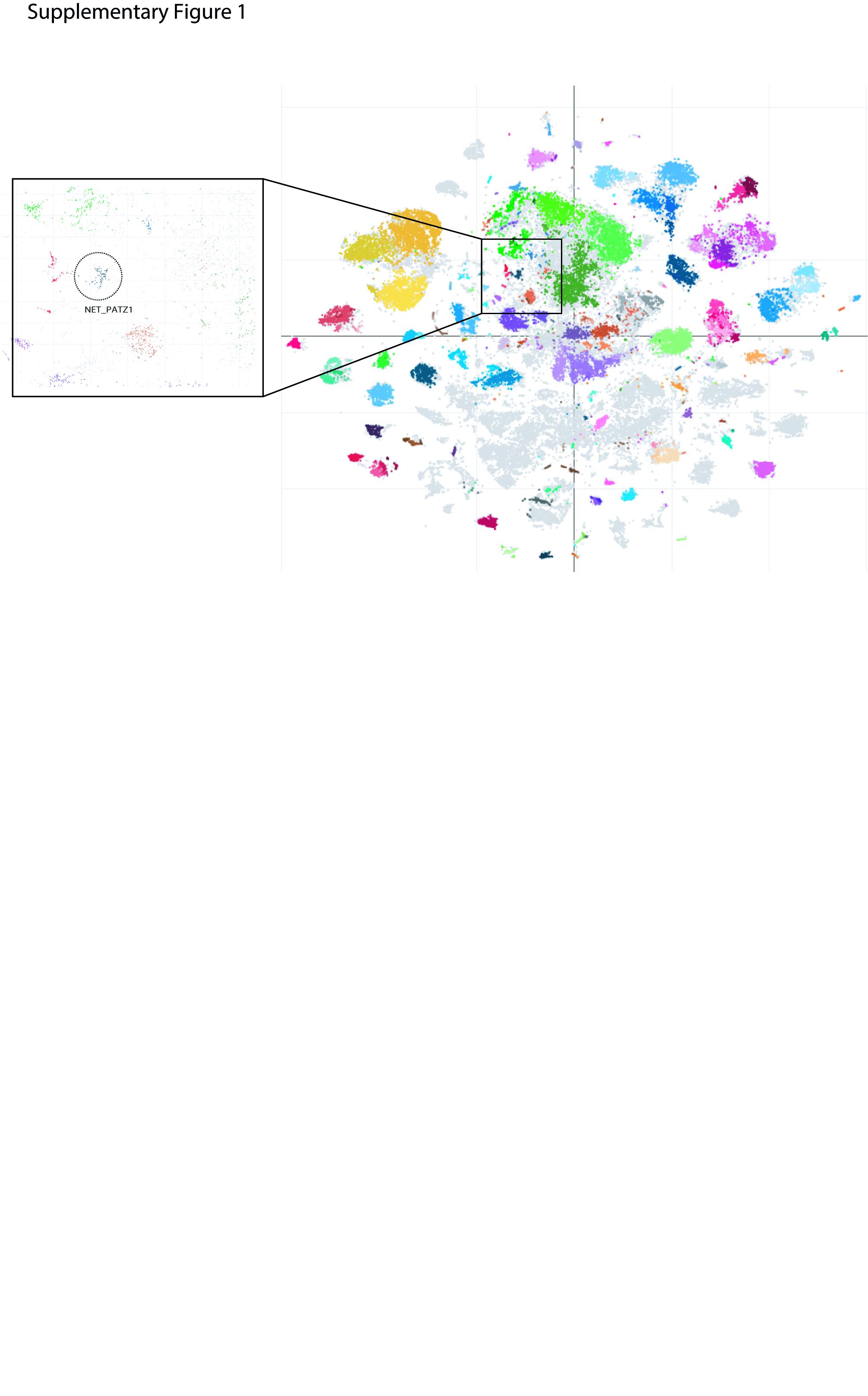

Supplement: Supplementary file 6 — Supplementary file6. Supplementary Fig. 4 a PATZ1-013: marked perivascular hyalinization and microcysts are seen together with monomorphous nuclei and small cells arranged in lobules. b PATZ1-056 displayed spindle-shaped cells with strong resemblance to a mesenchymal phenotype. c Immunohistochemistry of available sections for PATZ1-056 revealed very sparse staining with antibodies against GFAP and negative staining for Olig2, synaptophysin and MAP2, in keeping with the gene expression-based analysis. Scale bars represent 100 µm. Supplementary Table 1 includes more information about the staining patterns seen in NET_PATZ1 (TIF 39143 kb) [file 401_2021_2354_MOESM6_ESM.tif]
